# The Effects of Intravermis Cerebellar Microinjections of Dopaminergic Agents in Motor Learning and Aversive Memory Acquisition in Mice

**DOI:** 10.3389/fnbeh.2021.628357

**Published:** 2021-02-25

**Authors:** Evelyn M. Guilherme, Anna Carolyna L. Gianlorenço

**Affiliations:** Laboratory of Neuroscience, Department of Physical Therapy, Center of Biological Sciences and Health, Federal University of São Carlos, São Carlos, Brazil

**Keywords:** cerebellum, dopaminergic agents, avoidance learning, motor activity, motor learning

## Abstract

The cerebellum receives dopaminergic innervation and expresses the five types of described dopaminergic receptors. The cerebellar function involves both motor movement and cognition, but the role of cerebellar dopaminergic system on these processes remain unclear. The present study explores the behavioral responses to intracerebellar microinjection of dopaminergic agents in motor and emotional memory. For this, naïve Swiss mice had their cerebellar vermis implanted with a guide canula, received a intravermis microinjection of Dopamine, D1-like antagonist SCH-23390 or D2-like antagonist Eticlopride, and underwent a behavioral analysis of motor learning (by a Rotarod and balance beam learning protocol) or aversive memory acquisition (by the inhibitory avoidance task). The mixed-effects analysis was used to evaluate groups performance, followed by Tukey’s *post hoc* when appropriated. In this study, Dopamine, SCH-23390 and Eticlopride at the doses used did not affected motor control and motor learning. In addition, the administration of Dopamine and SCH-233390 had no effects on emotional memory acquisition, but the animals that received the highest dose of Eticlopride had an improvement in aversive memory acquisition, shown by a suppression of its innate preference for the dark compartment of the inhibitory avoidance apparatus following an exposure to a foot shock. We propose that cerebellar dopaminergic D2 receptors seem to participate on the modulation of aversive memory processes, without influencing motor performance at the doses used in this study.

## Introduction

Dopamine is a biogenic amine derived from hydroxylation and decarboxylation of tyrosine (Rodwell, 2003), and its most prominent neuronal portion is located in the ventral midbrain, sending projections to multiple areas of the central nervous system (CNS) (Chinta and Andersen, 2005). There are five characterized types of dopaminergic receptors (D1, D2, D3, D4, and D5), which are classified into two subgroups, according to its structural and functional similarities: D1-like (D1 and D5), and D2-like (D2, D3, and D4) (Strange, 1993; Jaber et al., 1996). Dysregulations of dopaminergic metabolism can lead to several diseases, such as Parkinson’s disease, epilepsy (Star, 1996), and schizophrenia (Dahoun et al., 2017). Hence, comprehending dopamine signaling pathways is the first step to discovery new therapies for these diseases (Klein et al., 2019).

The dopaminergic system is known to play a role in learning and memory processes in the prefrontal cortex (PFC) (Puig et al., 2014), anterior cingulate cortex, basolateral amygdala (Zheng et al., 2008), and hippocampus (Hamilton et al., 2010). However, despite the dopaminergic innervation (Panagopoulos et al., 1991) and expression of the five types of dopaminergic receptors in the cerebellum (Barili et al., 2000) its role on modulation of motor and emotional mnemonic processes is unclear.

The cerebellum has been traditionally associated with movement control but also it has recently related to cognition modulation (Koziol et al., 2014; Adamaszek et al., 2017), including learning and memory processes (Strata, 2015; Caligiore et al., 2019). The cerebellar vermis seems to represent an interface between sensorial stimuli, emotional processing, and motor responses, as a result of its connection with several important structures for the processing of such functions (Sacchetti et al., 2009). In a consensus article by Koziol et al. (2014), the authors suggest that the cerebellum acts establishing internal models for the coordination of movement and thought, modulating behavioral outcomes. Leggio and Molinari (2015) hypothesized that these internal patterns can be used to predict future components such as the body and the environment, explaining the cerebellar participation in learning processes.

Little is known about the role of cerebellar dopaminergic agents on the modulation of motor and non-motor function. Some studies showed the involvement of D1-like receptors on memory processes: Myslivev̧ek et al. (2007) observed an worsened spatial learning after i.p. administration of the D1 receptor antagonist SCH-23390 in mice with olivocerebellar degeneration, Locke et al. (2018) showed that the inhibition of D1 receptors of the lateral cerebellar nucleus (LCN) results in decreased spatial and working memory, and Heskje et al. (2020) suggests that the stimulation of D1 receptors of LCN may modulate frontal cortex circuitry and processing. Regarding the D2-like receptors, studies have focused on its motor role, showing an immediate decrease on spontaneous movement after intracerebellar administration of a D2-like agonist (Boulay et al., 2000; Kolasiewicz and Maj, 2001; Barik and Beaurepaire, 2005; Kolasiewicz and Ossowska, 2008; Shimizu et al., 2014).

Hence, we investigated how the intravermis infusion of Dopamine, D1-like receptor antagonist SCH-23390, and D2-like receptor antagonist Eticlopride influences in mice behavior during motor learning and inhibitory avoidance protocols. Based on the cerebellar dopaminergic innervation and on the important role of the cerebellum on processing and modulating motor and non-motor functions, we expect that such manipulation can modify both behaviors, and provide initial insights on the role of the cerebellar dopaminergic system in learning and memory.

## Materials and Methods

### Animals

The experimental subjects were 201 male Swiss mice (Federal University of São Carlos – UFSCar, SP, Brazil), from 35 to 50 days old, weighing 25–35 g at testing. The mice were housed in groups of five animals per cage (31 cm × 20 cm × 13 cm) and maintained under a 12 h light cycle (lights on at 7:00 a.m.) in a controlled environment at a temperature of 23 ± 1°C and humidity of 50 ± 5%. All mice were experimentally naïve at the beginning of the study. The experimental sessions were conducted during the light period of the cycle (8:00 am – 4:00 pm) to minimize the influence of the circadian rhythm on behavioral responses.

This study was approved by the Animal Ethics Commission of the Federal University of São Carlos (protocol 4486110220), which follows the standards of the Brazilian Neuroscience and Behavior Society (SBNeC), based on the US National Institutes of Health Guide for Care and Use of Laboratory Animals.

### Drugs

Dopamine, SCH-23390 (D1 antagonist) and Eticlopride (D2 antagonist) (Sigma Chemical Co., St. Louis, MO, United States) were prepared in sterile 0.9% saline solution (SAL). The Dopamine solution was prepared at doses of 0.29, 0.86, and 1.5 nmol/0.1 μl; SCH-23390 at doses 0.31, 0.92, and 1.54 nmol/0.1 μl; and Eticlopride at doses 0.26, 1.32, and 2.65 nmol/0.1 μl. The solutions were stored in coded tubes until the microinjection, and the experimenter was blinded to the codes during behavioral and statistical analysis.

We selected doses that presented effects in previous studies with different types of behavioral protocols. For instance, Nasehi et al. (2016) performed intra BLA injections of SCH-23390 at doses 0.01, 0.1 and 0.5 μg/mouse, and observed that the higher dose of 0.5 μg (1.54 nmol) impaired memory acquisition at the passive avoidance test, but did not have any effect on locomotor activity. Boye et al. (2001) observed that intra-accumbens infusion of eticlopride at doses 0.25 and 0.5 μg resulted in inhibition of spontaneous activity, and the highest dose of 1.0 μg produced evidence of antagonism with nicotine, reducing locomotor activity.

### Surgery and Microinjection

Mice received a general anesthesia with ketamine hydrochloride (100 mg/kg, IP) and xylazine (10 mg/kg, IP), and local anesthesia on the scalp (3% lidocaine with norepinephrine; 1:50.000), and then placed in a stereotaxic instrument. Mice cerebellar vermis (lobules 4-5) were implanted with a single 7-mm guide cannula (25-gauge; Insight Equipamentos Científicos, Brazil), according to the following coordinates from the mouse brain atlas of Franklin and Paxinos (2001): 6.5 mm posterior to the Bregma; 0 mm lateral to the midline; and 2.0 mm ventral to the skull surface. The guide cannula was fixed to the skull using dental acrylic (Blue dent, Brazil) and jeweler’s screws. A dummy cannula (33-gauge) was inserted into the guide cannula to reduce the incidence of occlusion. Postoperative analgesia was provided by adding acetaminophen (200 mg/ml) to the drinking water at a ratio of 0.2 ml acetaminophen to 250 ml water for a final concentration of 0.16 mg/ml.

On the third day post surgery (Curzon et al., 2009), saline or drug solutions were infused into the cerebellar vermis using a microinjection unit (33-gauge cannula; Insight Equipamentos Científicos Ltda, Brazil), which was attached to a 5 μl Hamilton micro syringe via polyethylene tubing, and an infusion pump that was programmed to deliver a volume of 0.1 μl over 60 s. Each animal received a microinjection of a single dose of the drug, according to the experimental group that they were randomly allocated.

### Apparatus

#### Rotarod

The Rotarod apparatus consists of a dark acrylic box (450 mm × 540 mm × 350 mm) with an 8 cm diameter non-slip cylinder, transversely installed approximately 20 cm from the floor of the equipment. The box is divided into five bays (8 cm length each), allowing the analysis of five animals simultaneously. The cylinder rotation was driven by a motor in a pre-set acceleration, that might be set at any speed from 0 to 50 rpm. In this study, it ranged from speed 8 to 20 rpm within 5 min in an incremental test. The falling latency was automatically measured by a sensor located on the floor of the device.

#### Balance Beam

The Balance Beam is a wood beam (100 x 2.8 cm) with a flat narrow surface (0.6 cm), resting 50 cm above the countertop on two acrylic poles. A dark box containing nesting material from home cages was placed at the finish point of the beam. A nylon hammock was installed bellow the beam to cushion any possible fall. The time spent for crossing the beam central 80 cm was automatically measured by two motion detectors (fabricated by Visopia) placed at the start and finishing points of the beam.

#### Inhibitory Avoidance Apparatus

The apparatus consists in an acrylic box (48 × 24.5 × 25 cm) divided into two equally sized compartments: one light (under illumination of 450 lux), and one dark (covered with black acrylic), separated by a guillotine door (9 × 10 cm). The floor is made of stainless-steel rods (2.5 mm in diameter), spaced 1 cm apart, that delivers electric shocks of 1.5 mA for 5 s. The guillotine door and the shock delivery are triggered by a connected computer software (Insight Equipamentos Cientificos Ltda., Brazil).

### Experimental Procedures

This study presents two distinct experimental procedures. The motor control and motor learning were assessed by the Rotarod and Balance Beam apparatus, and the aversive memory acquisition was assessed by the inhibitory avoidance apparatus. Each animal underwent only one experimental procedure, as described above.

#### Motor Control and Motor Learning

**FIGURE 1:**
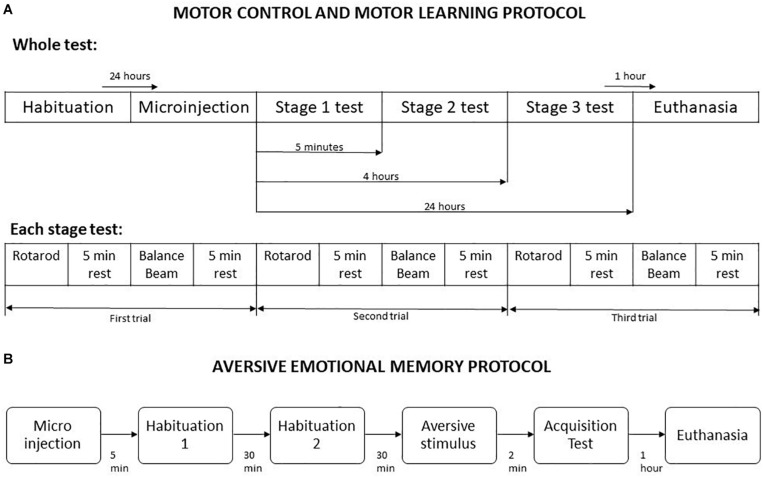
Schematic representation of behavioral testing procedures. **(A)** Motor control and motor learning protocol. **(B)** Aversive emotional memory acquisition protocol. Adapted from Song et al. (2006).

The combined use of Rotarod and Balance Beam allows the investigation of gross motor function and fine motor coordination (Curzon et al., 2009). This protocol was based on Song et al. (2006) and He et al. (2014) methodologies, with some modifications. It was divided into five steps, named habituation, microinjection, stage 1, stage 2, and stage 3. At habituation the animals were placed one time in the Rotarod apparatus up to 2 min or until they fall, and in the balance beam until crossing, which allowed the first contact of the animals with the apparatus. Twenty-four hours later, the microinjection procedure was performed using saline or one of the drugs doses, according to the experimental group. The stages 1, 2, and 3 were performed 5 min, 4 h, and 24 h after microinjection, respectively (Song et al., 2006). These timepoints permits the measurement of different motor learning processes, such as acquisition, and consolidation. For each stage, the animals were placed in the Rotarod in a crescent speed (8 to 20 rpm) up to 5 min or until falling, and in the balance beam until crossing. A five minutes interval was given between every exposure to the Rotarod and the balance beam. Overall, each animal performed three trials in Rotarod and three trial in balance beam per stage, totaling 9 trials per apparatus in the entire protocol (Figure 1). A mean of their scores in each stage was used in statistical analysis.

#### Aversive Memory Acquisition

The protocol started 5 min after the animals received a microinjection of saline or drugs, and was divided into habituation, aversive stimulus and memory acquisition test. In all stages, the mice were placed in the light compartment of the inhibitory avoidance apparatus, and the crossing latency to dark compartment was measured. For habituation, the crossing from the light side to dark compartment was allowed without any aversive stimulus, twice with a 30 min interval. In the next stage, the mice received a foot shock of 1.5 mA for 5 s as soon as they have crossed to dark compartment of the apparatus. The aversive memory acquisition test was performed 2 min after the foot shock delivery (Figure 1).

### Histology

**FIGURE 2:**
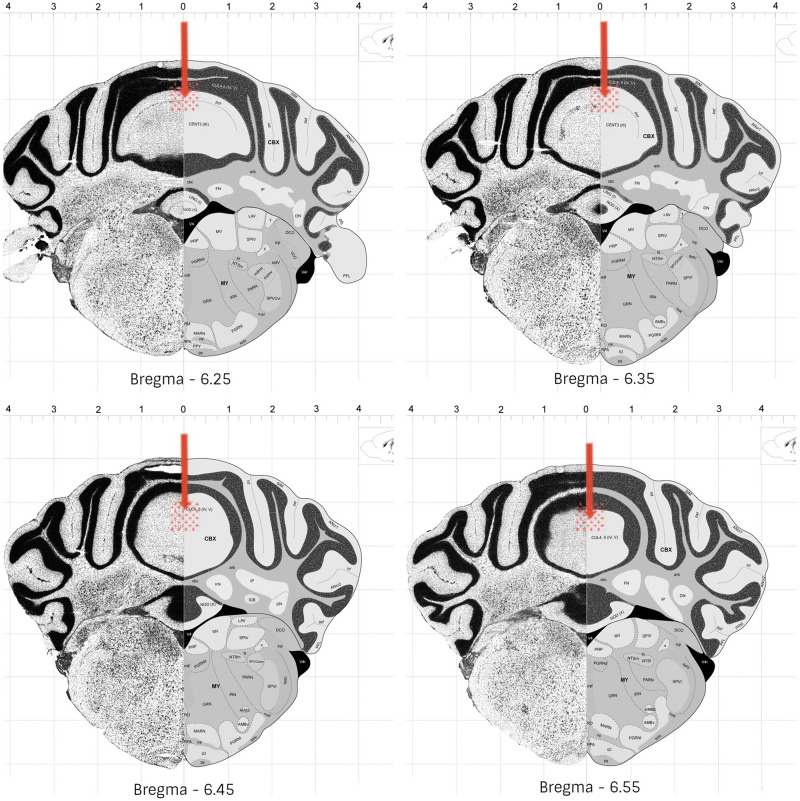
Schematic representation of the most common cannula placement (represented by arrow) and dispersion areas (represented by filled circles), in mice cerebellar vermis (lobules 4-5). Adapted from © 2008 Allen Institute for Brain Science. The Allen Mouse Brain Atlas. Available from: http://mouse.brain-map. org/static/atlas.

Following 60 to 90 min the end of the experiments, the animals received an anesthetic overdose, and were perfused transcardially with fixative (4% paraformaldehyde in 0.1 M phosphate buffer). The brains were removed and kept overnight in fixative. Coronal slices of 50 μm were cut with a vibratome and the injection sites were verified histologically according to the atlas of Franklin and Paxinos (2001). Animals with injection sites outside the cerebellar vermis were excluded from the study. Histological analysis confirmed that a total of 201 mice exhibited accurate positioning of the cannula placements in the vermal region of cerebellar lobules 4–5, mostly between bregma −6.25 and −6.55 (Figure 2).

### Statistical Analysis

The statistical analysis was performed using GraphPad Prism version 9.0.0 for Windows, GraphPad Software, San Diego, California, United States. Levene’s test confirmed homogeneity of variance. The ROUT method (Q = 1%) was used to identify extreme outliers. The mixed-effects analysis was used to evaluate groups performance. The within-subjects effect was measured by each exposure over time, and the between-subject factor was represented by the different groups of drug injection. Differences indicated by significant *P* values were further verified by *post hoc* Tukey’s multiple range test. In all cases, *p* < 0.05 was considered significant.

## Results

### Effects of Intravermis Cerebellar Microinjections of Dopamine on Motor Control and Motor Learning in Mice

**FIGURE 3:**
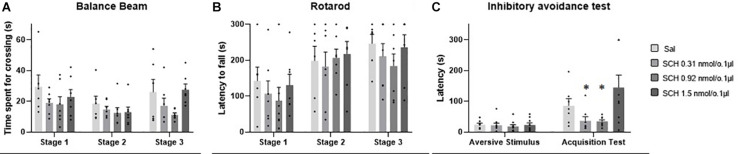
Scatter plot and bar graph of the effects of intravermis cerebellar microinjections of Dopamine at different doses on motor learning and emotional memory acquisition in mice. **(A)** Balance beam. **(B)** Rotarod. **(C)** Inhibitory avoidance test.

No outlier was identified in the Rotarod analysis, and nine outlier measurements were identified and removed from balance beam experimental group. The statistical analysis revealed that all groups presented an increase of the latency to fall of the Rotarod (*F*_1.842, 49.74_ = 1.39; *p* < 0.0001), and a decrease of time spent for crossing the balance beam (*F*_1.928, 43.37_ = 4.18; *p* = 0.02) through the three stages. However, there were no differences between the groups that received Dopamine in different doses and the control group, for the rotarod (*F*_3,27_ = 1.74; *p* = 0.27), and balance beam (*F*_3,27_ = 1.74; *p* = 0.18) exposures over the stages, indicating that microinjection into the cerebellar vermis of Dopamine at doses 0.29, 0.86, and 1.5 nmol/0.1 ul did not show significant behavioral effects on gross motor function and fine coordination performance and learning in mice in this study (Table 1 and Figure 3).

**TABLE 1:** Effects of intracerebellar microinjections of Dopamine (DOP), SCH-23390 (SCH), and Eticlopride (ETI) at different doses in the falling latency of the rotarod, and time spent for crossing the balance beam of mice exposed to motor control and motor learning behavioral protocol.

		Stage 1		Stage 2		Stage 3	
Groups	N	Rotarod (s)	Balance beam (s)	Rotarod (s)	Balance beam (s)	Rotarod (s)	Balance beam (s)
SAL	7	36.77 ± 13.73	41.38 ± 19.62	107.36 ± 42.60	36.14 ± 6.21	149.35 ± 34.73	26.51 ± 12.0
DOP 0.29	8	54.22 ± 38.26	35.35 ± 12.78	95.92 ± 22.34	20.87 ± 7.41	182.17 ± 35.91	20.60 ± 6.45
DOP 0.86	8	41.77 ± 7.42	22.1 ± 8.15	118.62 ± 23.69	9.73 ± 1.58	179.46 ± 28.97	15.91 ± 4.65
DOP 1.5	8	84.05 ± 17.50	17.11 ± 2.96	178.21 ± 21.95	9.43 ± 1.3	209,0 ± 27.78	11.85 ± 2.52
SAL	6	142.58 ± 38.39	29.47 ± 7.68	198.59 ± 40.82	18.54 ± 4.83	246.9 ± 25.57	26.06 ± 8.22
SCH 0.31	7	103.28 ± 32.38	18.91 ± 2.65	182.32 ± 40.75	14.6 ± 2.06	211.30 ± 35.08	17.22 ± 4.74
SCH 0.92	7	96.41 ± 39.74	20.95 ± 4.56	223.99 ± 10.01	12.08 ± 3.73	195.61 ± 34.68	10.54 ± 1.12
SCH 1.54	7	130.7 ± 30.47	22.83 ± 4,81	218.15 ± 34.29	13.0 ± 3.27	236.39 ± 34.81	27.59 ± 3.64
SAL	8	130.98 ± 24.81	14.91 ± 2.41	190.37 ± 35.70	12.92 ± 2.70	241.85 ± 35.64	12.83 ± 2.20
ETI 0.26	8	117.28 ± 35.4	17.68 ± 2.94	208.29 ± 36.65	12.99 ± 1.83	235.33 ± 33.46	10.22 ± 1.93
ETI 1.32	8	108.59 ± 24.71	18.90 ± 2.93	182.54 ± 32.89	16.95 ± 3.86	197.57 ± 45.24	18.46 ± 2.14
ETI 2.65	9	66.73 ± 22.39	29.91 ± 7.4	173.85 ± 31.21	24.31 ± 8.43	227.18 ± 31.16	21.31 ± 8.59

*Mean ± SEM.*

### Effects of Intravermis Cerebellar Microinjections of Dopamine on Emotional Memory Acquisition in Mice

One outlier was identified and removed of this experimental group. The within groups comparison revealed that all groups showed appropriate aversive memory acquisition by an increase in crossing latency to the dark side of the inhibitory avoidance apparatus after the aversive stimulus (*F*_1,37_ = 54.12; *p* < 0.0001). However, there was no difference for the crossing latency among the groups that received Dopamine in different doses and the control group (*F*_3,38_ = 2.35; *p* = 0.09), which indicates that the microinjection in the cerebellar vermis of Dopamine in the doses 0.29, 0.86, and 1.5 nmol/0.1 ul had no significant behavioral effects on the emotional memory acquisition in mice in this study (Table 2 and Figure 3).

**TABLE 2:** Effects of intracerebellar microinjections of Dopamine (DOP), SCH-23390 (SCH), and Eticlopride (ETI) at different doses in the crossing latency do the dark side of the inhibitory avoidance apparatus of mice exposed to emotional memory acquisition behavioral protocol.

	N	Habituation 1 (s)	Habituation 2 (s)	Aversive stimulus (s)	Acquisition test (s)
SAL	8	20.44 ± 3.91	27.41 ± 4.14	21.81 ± 5.01	115.54 ± 22.40
DOP 0.29	14	46.56 ± 12.80	23.01 ± 4.92	29.02 ± 5.87	77.54 ± 19.29
DOP 0.86	9	51.54 ± 15.01	26.45 ± 5.14	23.18 ± 4.73	81.93 ± 12.45
DOP 1.5	11	33.51 ± 9.53	20.62 ± 4.28	14.73 ± 1.58	61.73 ± 14.69
SAL	7	29.05 ± 6.91	27.62 ± 6.83	24.13 ± 5.26	86.16 ± 22.59
SCH 0.31	9	34.84 ± 7.56	18.09 ± 6.45	22.23 ± 7.38	65.77 ± 31.47
SCH 0.92	10	27.29 ± 17.35	20.26 ± 4.92	18.43 ± 5.23	61.15 ± 26.94
SCH 1.54	9	3.95 ± 11.54	14.19 ± 3.63	23.59 ± 6.64	144.96 ± 40.51
SAL	9	53.66 ± 10.39	29.50 ± 3.79	24.04 ± 4.38	102.17 ± 13.61
ETI 0.26	9	54.17 ± 15.71	25.10 ± 3.95	16.90 ± 5.90	102.27 ± 30.85
ETI 1.32	8	25.64 ± 5.12	28.0 ± 4.88	35.61 ± 10.41	122.96 ± 21.79
ETI 2.65	7	36.11 ± 5.61	39.22 ± 7.35	46.76 ± 5.20	199.91 ± 28.96*

*Mean ± SEM.*

### Effects of Intravermis Cerebellar Microinjections of D1-Like Antagonist on Motor Control and Motor Learning in Mice

**FIGURE 4:**
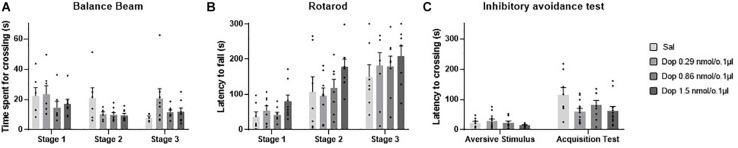
Scatter plot and bar graph of the effects of intravermis cerebellar microinjections of D1-like antagonist SCH-23390 at different doses on motor learning and emotional memory acquisition in mice. **(A)** Balance beam. **(B)** Rotarod. **(C)** Inhibitory avoidance test. *Significant difference from SCH-23390 at 1.5 nmol/0.1 ul (*p* < 0.001).

No outlier was identified in the rotarod and balance beam experimental groups. The statistical analysis indicated that all groups presented an increase of the latency to fall of the Rotarod (*F*_1.990, 45.77_ = 20.39; *p* < 0.0001) and a decrease of time spent for crossing the balance beam (*F*_1.494, 34.36_ = 4.12; *p* = 0.03) through the three stages. However, there were no significant differences between the groups that received the D1-like receptor antagonist SCH-23390 in different doses and the control group in mice exposed to rotarod (F_3,23_ = 0.44, *p* = 0.72) and the balance beam (*F*_3,23_ = 2.16; *p* = 0.12). These results show that the intra vermis cerebellar microinjection of SCH-23390 did not promote significant changes in the motor performance and motor learning in mice at the doses used in this study (Table 1 and Figure 4).

### Effects of Intravermis Cerebellar Microinjections of D1-Like Antagonist SCH-23390 on Emotional Memory Acquisition in Mice

Two outlier measurements were identified and removed from this experimental group. The within groups comparison revealed that all groups presented an increase in crossing latency to the dark side of the inhibitory avoidance apparatus after the aversive stimulus (*F*_1,29_ = 21.68; *p* < 0.0001). Moreover, a significant difference for the crossing latency was found between groups (*F*_3,31_ = 4.07; *p* = 0.01). The multiple comparisons test revealed that there were no differences between the control group and the treated groups; however, the groups that received the D1-like antagonist SCH-23390 at the lower doses of 0.31 and 0.92 nmol/0.1 ul presented a lower crossing latency than the group that received the higher dose of 1.54 nmol/0.1 ul (*p* = 0.0004; *p* = 0.0002, respectively) (Table 2 and Figure 4).

### Effects of Intravermis Cerebellar Microinjections of D2-Like Antagonist Eticlopride on Motor Control and Motor Learning in Mice

**FIGURE 5:**
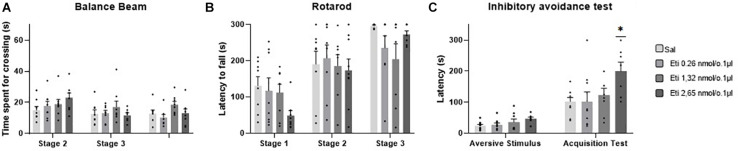
Scatter plot and bar graph of the effects of intravermis cerebellar microinjections of D2-like antagonist Eticlopride at different doses on motor learning and emotional memory acquisition in mice. **(A)** Balance beam. **(B)** Rotarod. **(C)** Inhibitory avoidance test. *Significant difference from saline control group (*p* = 0.002), Eticlopride 0.26 nmol/0.1 μl group (*p* = 0.002), and Eticlopride 1.32 nmol/0.1 μl group (*p* = 0.2).

Five outlier measurements were identified and removed from the rotarod, and four outliers were removed from balance beam experimental group. The statistical analysis revealed that all groups presented an increase of the latency to fall of the Rotarod (*F*_1.993, 52.83_ = 53.54; *p* < 0.0001), and a decrease of time spent for crossing the balance beam (*F*_1.847, 49.86_ = 7.53; *p* = 0.001) through the three stages. No significant differences were found between the groups that received the D-2 like antagonist Eticlopride and the control group, for the behavioral tests in Rotarod (*F*_3,29_ = 0.49; *p* = 0.69) and balance beam (*F*_3,29_ = 1.15; *p* = 0.35). These data suggest that microinjection into the cerebellar vermis of Eticlopride at doses 0.26, 1.32, and 2.65 nmol/0.1 ul did not lead to significant changes in the motor performance and motor learning in mice in this study (Table 1 and Figure 5).

### Effects of Intravermis Cerebellar Microinjections of D2-Like Antagonist Eticlopride on Emotional Memory Acquisition in Mice

No outlier was identified in this experimental group. Regarding the emotional memory acquisition, there was a significant difference observed within-subjects in all groups exposed to inhibitory avoidance protocol (*F*_1,29_ = 64.23; *p* < 0.0001). Moreover, the statistical analysis revealed that there were differences of crossing latency between-subjects (*F*_3,29_ = 4.15; *p* = 0.01), and Tukey’s *post hoc* showed that the group that received the higher dose of the D2-like antagonist Eticlopride (2.65 nmol/0.1 μl) presented a significant increase in the latency of crossing to the dark side of the inhibitory avoidance apparatus when compared to control group (*p* = 0.002) and the groups that received Eticlopride at 0.26 nmol/0.1 μl (*p* = 0.002) and at 1.32 nmol/0.1 μl (*p* = 0.02). These data suggest that the intracerebellar microinjection of Eticlopride at the dose 2.65 nmol/0.1 μl improved aversive memory in mice exposed to inhibitory avoidance protocol in this study (Table 2 and Figure 5).

## Discussion

In this study, we found that mice that received an intracerebellar microinjection of the D2-like antagonist Eticlopride at dose 2.65 nmol/0.1 ul had an improvement in aversive emotional memory, by a suppression of its innate preference for the dark compartment of the inhibitory avoidance apparatus following an exposure to a foot shock. However, the lower doses of Eticlopride, and the microinjection of Dopamine and D1-like antagonist SCH-23390 had no significant behavioral effect at the same test, that has been widely used to evaluate aversive emotional memory in rodents (Gold, 1986; Izquierdo and Medina, 1997; Arakawa, 2019).

Previous studies have shown that dopaminergic signaling is critical for emotional memory formation (Saito et al., 2020; Steinberg et al., 2020). In a review article, Likhtik and Johansen (2019) showed that dopaminergic neurons projects from different midbrain regions, including ventral tegmental area (VTA) and substantia nigra (SN), to lateral and central amygdala, modulating fear learning by responding to unexpected events and cues that predict them. At the same time, VTA sends dopaminergic projections to the cerebellar cortex (Ikai et al., 1996) and receives projections from Purkinje cells (Snider et al., 1976) and cerebellar nuclei (Watabe-Uchida et al., 2012; Carta et al., 2019), which might be an important path by which the cerebellum modulates information from other limbic-related structures (Ikai et al., 1994). Moreover, the cerebellum seems to be able to exert inhibitory control (Gil-Miravet et al., 2019) and modulate dopamine levels in the mPFC (Rogers et al., 2011), a key structure on the modulation of aversive memories (Canto-de-Souza and Mattioli, 2016), which in turn control the VTA dopaminergic systems that innervates amygdala and hippocampus (Izquierdo et al., 2016). In this way, we believe that the complex cerebellar-VTA-PFC connections might be a key element for dopaminergic modulation of aversive memory.

It is known that an increase in the cerebellar vermis activation occurs in face of an aversive stimulus (Ernst et al., 2019), but there is not enough information about how the cerebellar dopaminergic system acts on the modulation of memory processes. A recent study mapped the tyrosine hydroxylase – a dopamine precursor – at several cerebellar regions, including the posterior vermis, showing that catecholaminergic signaling, within a subset of Purkinje cerebellar cells, can modulate fear conditioning without affecting gross motor function on accelerating rotarod (Locke et al., 2020). Based on these findings, and in the results of the present study, we believe that the dopaminergic system – more specifically the D2-receptor - may play a role in the cerebellar modulation of the emotional memory.

Despite the Dopamine and D1-like antagonist SCH-23390 microinjections did not induce significant changes in the aversive memory acquisition in this behavioral test, its effects should not be neglected. The highest dose of Dopamine lead to an approximately 46% decrease in the crossing latency mean compared to control group, whereas the highest dose of SCH-23390 caused an 40% increase for the same variable compared to controls. On that basis, the present results do not entirely rule out the possibility of a D1-receptor role on the modulation of aversive memory acquisition.

Regarding the role of dopaminergic agents on motor learning, we found that the intracerebellar microinjections at different doses of Dopamine, D1-like receptor antagonist SCH-23390, and D2-like receptor antagonist Eticlopride had no significant effects on motor performance at the rotarod and balance beam behavioral tasks through the three stages learning protocol.

Some studies have demonstrated the dopaminergic role on motor performance and motor learning. For instance, the i.p. administration of D1-like antagonists have been related to deficits in motor coordination (Avila-Luna et al., 2016), and the absence of D2 receptors leads to severe impairments in motor coordination, locomotion, and motor learning (Bello et al., 2017; Lim et al., 2019). Furthermore, The D1 and D2 antagonists administered in the motor cortex impairs motor skill acquisition and synaptic plasticity (Molina-Luna et al., 2009; Rioult-Pedotti et al., 2015). However, the dopaminergic modulation of the cerebellar motor function is not clear.

As mentioned at the introduction section, some studies observed an immediate decrease on spontaneous movement after the administration of a D2-like agonist into the cerebellar lobules 9-10 (Kolasiewicz and Maj, 2001; Barik and Beaurepaire, 2005; Kolasiewicz and Ossowska, 2008; Shimizu et al., 2014), and no studies were found regarding the influence of dopaminergic agents in cerebellar motor learning. However, according to Fujita et al. (2020), the vermis presents several modules across its multiples lobules, which can be linked with brainstem nuclei in different ways, sub serving a variety of functions. We assume that the vermal region observed in the present study is not related to motor coordination and motor learning processes, but to the cognitive functions such as the passive avoidance, explaining the results of this study.

In conclusion, we believe that the cerebellum has a role as a modulator in adaptative behavior, such as the passive avoidance acquired after an aversive stimulus. Our view is that the cerebellum – by its connections with other brain structures and using its dopaminergic projections – might promote behavioral adjustments in similar ways of its mechanisms for adjusting voluntary movements. However, the specific cerebellar pathways involved in aversive emotional memory acquisition needs further investigation.

## Study Limitations

This study has potential limitations. Despite some of the results did not reach statistical significance, a possible practical relevance of the drugs administration must be taken into consideration. A complete behavioral testing battery could provide elucidating data regarding the effects of dopaminergic system in such functions. Moreover, there is a lack of previous studies focusing on intracerebellar dopaminergic compounds administration, making the right drug dosage selection difficult. Further research should focus on the effects of different drug doses, providing a complete dose-dependent effect in learning and memory functions.

## Data Availability Statement

The raw data supporting the conclusions of this article will be made available by the authors, without undue reservation.

## Ethics Statement

The animal study was reviewed and approved by Animal Ethics Commission of the Federal University of São Carlos, which follows the standards of the Brazilian Neuroscience and Behavior Society (SBNeC).

## Author Contributions

EG and AG designed the experiments. EG conducted the behavioral experiments, analyzed the data, and wrote the manuscript. AG reviewed and edited the manuscript. Both authors contributed to the article and approved the submitted version.

## Conflict of Interest

The authors declare that the research was conducted in the absence of any commercial or financial relationships that could be construed as a potential conflict of interest.
